# Role-Play as Responsible Robotics: The Virtual Witness Testimony Role-Play Interview for Investigating Hazardous Human-Robot Interactions

**DOI:** 10.3389/frobt.2021.644336

**Published:** 2021-06-29

**Authors:** Helena Webb, Morgan Dumitru, Anouk van Maris, Katie Winkle, Marina Jirotka, Alan Winfield

**Affiliations:** ^1^ Department of Computer Science, University of Oxford, Oxford, United Kingdom; ^2^ Bristol Robotics Lab, University of West of England, Bristol, United Kingdom; ^3^ Royal Institute of Technology, Stockholm, Sweden

**Keywords:** role-play, interview, responsible research and innovation, methods, human-robot interaction

## Abstract

The development of responsible robotics requires paying attention to responsibility within the research process in addition to responsibility as the outcome of research. This paper describes the preparation and application of a novel method to explore hazardous human-robot interactions. The Virtual Witness Testimony role-play interview is an approach that enables participants to engage with scenarios in which a human being comes to physical harm whilst a robot is present and may have had a malfunction. Participants decide what actions they would take in the scenario and are encouraged to provide their observations and speculations on what happened. Data collection takes place online, a format that provides convenience as well as a safe space for participants to role play a hazardous encounter with minimal risk of suffering discomfort or distress. We provide a detailed account of how our initial set of Virtual Witness Testimony role-play interviews were conducted and describe the ways in which it proved to be an efficient approach that generated useful findings, and upheld our project commitments to Responsible Research and Innovation. We argue that the Virtual Witness Testimony role-play interview is a flexible and fruitful method that can be adapted to benefit research in human robot interaction and advance responsibility in robotics.

## Introduction

The COVID-19 pandemic has created massive disruption to research projects relying on data collection involving human participants. At the same time, the need for adaptation has fostered opportunities for creativity in the adoption and application of methods. This paper describes how our research team developed and tested a new approach that enabled us to explore human-robot interaction whilst working from home. We developed a research protocol to investigate accidents involving social robots and humans; this protocol uses an online format and invites human participants to role-play a scenario in which they become observers to the aftermath of an accident and provide witness testimony in relation to it. This research formed part of our ongoing project work on responsible robotics and, despite the constraints, the pandemic proved to be a catalyst for innovation in our approach and enabled us to work through certain logistical and ethical challenges we were facing in our study. The protocol we developed provided a means for us to incorporate principles of Responsible Research and Innovation (RRI) into our work by establishing a safe and ethical process through which participants can experience hazardous human-robot interactions. In this paper, we briefly outline the overall focus of our project and then catalogue the decision-making that led to the development of our new research protocol. We situate this approach within a discussion of role-play in studies of human-robot interaction (HRI) and human-computer interaction (HCI) more generally. We provide a detailed account of how our initial set of Virtual Witness Testimony (VWT) role-play interviews were conducted and our findings section focuses on what we discovered about the efficiency of the approach, the quality of the results it generated, and its limitations. In the Discussion section we comment on how the VWT role-play interview forms a flexible and fruitful method that can be adapted to benefit others working in HRI and seeking to advance responsibility in robotics.

## Background

### RoboTIPS: Developing Responsible Robotics for the Digital Economy

The ongoing RoboTIPS study (Webb et al., 2019) is a five-year fellowship project that explores opportunities for the development of responsible robotics within the context of the contemporary digital economy. The project is underpinned by a commitment to Responsible Research and Innovation (RRI), an initiative that seeks to ensure that processes of research and innovation benefit society and the environment (Stilgoe et al., 2013; Rome Declaration 2014). In the context of academic research, adopting an RRI approach involves acknowledging that the responsibilities held by researchers, universities and funders broaden out from traditional issues of research integrity and plagiarism etc., (vom Schomberg and Hankins 2019). This broadening brings in further aspects around research processes such as gender equality and stakeholder inclusion, and also requires attending to the social, policy and environmental impacts of work. Within this perspective, responsibilities for the practices and outcomes of research are shared out across the research ecosystem, research communities take on new co-responsibilities, and society (*via* stakeholders) becomes involved in research and innovation across all of its phases Owen et al., 2013.

A key strand of RoboTIPS examines the investigation of incidents and accidents involving social robots in which humans are harmed in some way (Winfield et al., 2021). We focus on social robots that interact with humans as part of their day-to-day function, in particular assistive robots, automated vehicles and robot toys. We take the position that as more and more robots become commercially available, incidents and accidents, whilst hopefully rare, can be expected to occur. Therefore, it is necessary to develop mechanisms to identify the causes of these incidents and take steps to prevent them re-occurring. Our project work includes the design, development and testing of an innovative safety feature for social robots. The Ethical Black Box (EBB) (Winfield and Jirotka, 2017) is a data recorder for social robots, equivalent to the flight data recorders used in aviation. It continuously records sensor and relevant internal status data and can be extended in scope to also capture the AI decision-making processes of the robot and environmental phenomena around it. Just as black boxes are used on in aviation to provide crucial evidence following an accident, so the EBB can be used as a data source following some kind of incident or accident involving a social robot. The information provided by the EBB can help to identify failures in the robot and to understand why it behaved in the way it did. This data is used as part of a wider investigation process. Human witnesses to the incident report their recollections and understandings of the event, and the EBB data provides another form of witness testimony. In addition, various experts provide details about the specific setting and the robots involved. As a result, this investigation process aims to determine the cause of the incident and then produce recommendations–which might take the form of technical changes to the robot and its setup, as well as organisational changes in the setting–to prevent similar events from occurring in future and therefore avoid further harms. In this way, the EBB-informed investigation process serves as an innovation for safety, trust, accountability, and transparency in social robotics.

### Incidents and Accidents Involving Social Robots: Investigating Hazardous Human-Robot Interactions

Our RoboTIPS project work to develop and trial the EBB requires us to understand how accidents involving social robots and humans unfold, and how humans at the scene respond to them. This includes understanding how humans might interact with the robot in the aftermath of an incident and how (as well as how much) they recall what they saw afterwards. Deriving this understanding will help us to optimise the accident investigation process, for instance in determining what kind of interactions humans might have with an EBB-enhanced robot in the context of an accident and how EBB data can best supplement testimony provided by human witnesses. We ultimately plan to run a series of laboratory-based simulated scenarios in which we stage an accident involving real robots and human participants and then run an investigation process with expert participants who will work through the human and robot witness data to discern the causes of the accident. This quasi-naturalistic approach will collect highly valuable data but also represents logistical and ethical challenges. It requires a great deal of advance planning and piloting to ensure it is fully workable: care needs to be taken to ensure that the accident scenario is viable and realistic but does not cause any actual harm. Careful organization is needed so that the processes of accident and subsequent investigation run smoothly in the time available, and all participants are required to give at least one full day of their time. In terms of ethical issues, observing a simulated scenario in which a human being appears to be physically harmed and at risk could potentially cause a research participant distress. Whilst participants will be aware that they are taking part in a research exercise and therefore that what they see was staged, it is possible that a realistic looking scenario might lead them to forget this momentarily and become upset at what they see. At this stage in the project, we do not know how much of a risk this is. In particular, due to the newness of robotic technology, we don’t know the extent to which the presence of a robot in a simulated accident scenario, coupled with the potential that it may cause harm to a human, might trigger participant discomfort or distress.

The switch to remote working necessitated by the United Kingdom lockdown in response to the spread of COVID-19 shifted our attention to the use of an online format for fieldwork. We realized that we could draw on this format to continue our work on accident scenarios but do so in a way that limited the logistical and ethical challenges outlined above. Specifically, we saw the benefit in asking our online participants to role-play a scenario in which they were witnesses to the occurrence or aftermath of an accident involving a social robot. Setting up and running the data collection would be relatively quick and non-labour intensive–especially in comparison to simulating the scenario in laboratory conditions. If the accident scenario proved to be unrealistic or the witnessing process unviable, we would have opportunities to make quick alternations and try again. We could use our participants’ responses to learn more about the process of witnessing accidents and also use them as testimony in accident investigation exercises in our study. In addition, the distance provided by an online platform, combined with the absence of actual robots, could create a safe space in which participants could experience hazardous interactions with a robot. We would be able to elicit their responses as if they were in the scenario, to learn about their interactions with the robot but with far less risk of making them feel uncomfortable or distressed. As such we would be putting our commitment to RRI into practice. We decided to develop a research protocol based on this online approach and trial it. As we demonstrate in this paper our trials show it to be a highly useful method. In RoboTIPS we plan to use it as a complement to (and preparation for) future laboratory-based simulations, but it can also be used as an alternative to *in situ* human-robot interaction studies. Before we describe the research protocol and its development, we spend some time discussing role-play as method and how it can contribute to the study of human-robot interaction.

### Role-Play as Research Method

Broadly speaking, the term “role-play” in research describes a multi-party interaction in which individuals play out a series of actions based on taking a specific role (Lewis-Beck et al., 2004). Individuals may take on the role of an imagined other in a role-play but might also act as themselves. The technique has been widely used a tool for communication skills training in medicine and beyond (Joyner and Young, 2006; Stokoe, 2014) as well as one for language learning (Ladousse, 1995). The aim of the research-focused role-play is to investigate how participants respond to certain activities or stimuli within the interaction. The method can provide a highly effective means to simulate a scenario which is perhaps too complex or risky to stage naturalistically whilst eliciting useful data. It can also be used to elicit participant responses regarding hypothetical futures and emerging technologies, so is therefore of significant potential benefit to fields such as human-robot interaction (HRI). We conducted a literature review of role-play in HRI and found numerous references to the term, alongside references to other adjacent terms. There is an absence of consistent usage across the literature but we can broadly characterize these terms as: :“scenarios” - a combination of physical context and task created to replicate a real life situation in which human participants may or may not be involved; “simulations” which tend to be virtual scenarios or physical role-plays where human participants are optional and, if they do exist, play themselves; “narrative interactions,” which tend to be role-plays with a pre-planned narrative arc; and “imaginaries,” which tend to be fictional situations that come from the imagination of participants with some prompting by researchers.

This cluster of methods has been used in HRI in a number of ways. Typically, human behavior and responses to a particular HRI scenario are captured and observed through HRI experiments. These are mostly conducted in physically-situated, video-based or virtual reality contexts. Our review of the literature identified role-play (and its associated forms) deployed as a capability of robots, as a method of teaching humans and robots, as method of prototyping, and as method of conducting research. The latter two are the most common forms. Where role-play in HRI has been used as a HRI prototyping method, this work is intended both to test the performance of a specific human-robot-task-context combination and to test the methodology. The results can provide valuable insight into the human experience of interacting with robots. Tonkin et al. (2018) used role-plays to test prototype behaviours of a PAL REEM humanoid-wheeled social robot in preparation for deployment in an airport. The role-plays were conducted in a lab, where visitors interacted with the robot and provided feedback to researchers. The findings helped the team develop their design methodology by providing a mechanism for quick, early-stage feedback. Koay et al. (2020) provided further evidence for the value of narrative-based prototyping for social robots. They used episodic, narrative role-plays to prototype home companion robots. Participants interacted with multiple embodiments of a single agent in a series of 1-h role-plays, held twice a week for a month. Each session began with a narrative introduction, after which participants interacted with the robot exclusively, enabling the authors to examine user acceptance of narratively-connected scenario and user-agent relationships after embodiment migrations.

When used as a research method within HRI, role-plays have been conducted to test out a much wider range of research questions. For instance, existing studies have use role-plays to: gather input during robot design protocols (Vallès-Peris et al., 2018); attempt to reproduce “observed real-world social interactions with a robot” (Liu et al., 2016); test the development of natural language user interfaces for robots with cognitive capabilities (Green et al., 2006); plus to explore opportunities and challenges around the collaboration between humans and robots in industrial settings (Meneweger et al., 2015; Weiss et al., 2016) and identify ways to optimize this collaboration (Wurhofer et al., 2015; Weiss and Huber 2016). Frequently these role-plays do not involve human participants interacting with actual robots; instead Wizard-Of-Oz style simulations are deployed instead. For instance, in their 2006 paper, “Measuring Up as an Intelligent Robot–On the Use of High-Fidelity Simulations for Human-Robot Interaction Research,” Green et al. ran two such simulations in which participants gave a robot a tour of a staged home environment and the robot’s actions were tele-operated. This proved a viable method for developing the social robots in the areas of spatial language research, whole-system conceptualization, and user attitude assessment. Staging role-plays without a real robot can help to protect vulnerable research participants. Vallès-Peris et al. (2018) used imaginaries to engage with first-grade aged school children and encourage them to share their needs, feelings and preferences around robots in healthcare contexts.

The literature on role-play in HRI research is informative but still relatively small. We were interested to note that we could not find any examples of role-play being conducted in an online format, with participants communicating over an online platform whilst physically distanced. This is a significant gap that neglects the potential of the remote format. We were also interested to look at other fields that have embraced role-play to see what we could learn about the value of the method as well as the challenges it presents. The use of role-play in areas such as education, entertainment, and design is highly illuminating here. Role-plays have been used in a wide range of formats and for a variety of purposes. For instance, physical role-plays with tokens have been used to teach farmers in Ghana (Villamor and Badmos, 2016) and virtual games have been used to teach cultural awareness to military operatives in the United States (Prasolova-Førland et al., 2013). MMORPGs like World of Warcraft have eclipsed their live-action and pen-and-paper predecessors in the realm of interactive entertainment. Designers have used research-oriented games to explore plausible futures (Coulton et al., 2016) and narrated scenarios to refine communication tool prototypes (Nielsen, 2012).

Examination of the literature reveals the importance of enabling participants to fully engage with the context presented in the role-play, by making it immersive or as realistic as possible, in order to elicit genuine responses from them. This is exemplified in Mariani (2020) paper, “Other Worlds. When Worldbuilding and Roleplay Feed Speculation,” which highlights the aspects of games that make them well suited for the exploration of alternative circumstances from an HCI perspective. The paper demonstrated that in allowing people to suspend their disbelief, the worldbuilding aspect of games makes them a valuable aid for “envisioning, speculating, and framing possibilities and alternatives.” Similarly, Ortiz and Harrell (2018) used their findings to argue that narrative role-plays were more effective than earlier HCI methodologies at facilitating engagement in the form of human self-reflection. Participants completed a virtual, single-party role-play, Chimeria:Grayscale via an online game the researchers had developed, and then answered a survey with system usability and game experience questions. The data from this survey suggested that Chimeria:Grayscale enabled self-reflection in participants and provided evidence for the authors’ ongoing research on computer-supported self-reflection.

A final set of work that proved very illuminating in informing our work also comes from HCI and concerns the use of role-play (and other naturalistic techniques) to facilitate cultural experiences. Benford et al. (2012), Benford et al. (2015) have conducted various studies to expose participants to unusual and often uncomfortable interactions. These take many forms but have included allowing participants to first watch a breath-powered swing ride and then take on the role of controller, determining another’s experiences, as well as participation in a large-scale community alternate reality game (ARG) in which participants observe a “kidnap” and sign up to be players in the game to investigate that crime with some of them ultimately being kidnapped themselves and being interrogated about what they knew. Although the scenarios are carefully designed to be physically safe, they are also designed to prompt feelings of thrill and excitement in the participant, which may tip over into discomfort or fearfulness. Benford and his collaborators (2015) argue that it is possible to conduct such work in ways that is immersive to participants in order to elicit genuine and spontaneous responses from them but that is also ethical and manages the risks involved. In some cases, this may involve the provision of consent from the participant through a process of negotiation across the encounter, rather than in an informed consent phase at the very beginning of it. This upholds the participants’ rights to determine their own experiences and to withdraw if they choose but also avoids the full nature of the experience being revealed early on and so preserves opportunities for more spontaneous responses as the situation unfolds.

Insights from these various literatures helped to inform our own study design. As described further in the next section, we adopted the use of a narrator-led role-play to facilitate participant interaction in a setting involving a social robot. This would occur online rather than in person - a necessity due to COVID-19 social distancing constraints but also an arrangement that was highly time efficient and placed minimal demand on participants. In this arrangement we needed to find ways to encourage our remote participants to engage with the setting presented to them. Since our project involves a focus on incidents and accidents, we were further interested in how we could explore hazardous or uncomfortable interactions between humans and robots and do so in a way that was safe and ethical. In the next section we our novel research approach, the Virtual Witness Testimony role-play interview, in detail.

## Methods: The Virtual Witness Testimony Role-Play Interview

As described above, we decided to conduct online interviews in which human participants role-played a response to an accident scenario. Specifically, we wanted our participants to witness the aftermath of an accident involving a social robot and a human so that we could then elicit their responses to it as if they were in that scenario and then elicit their recollections of it afterward. The work was designed to benefit our RoboTIPS study by providing insights into processes of human witnessing and thereby help us optimize the conduct of EBB-informed accident investigation processes. As a safe and efficient means to expose participants to hazardous human-robot interactions the approach can also be used on its own or–as we intend to do in RoboTIPS–as a complement to further, more naturalistic, methods.

### Development of the VWT Role-Play Interview

We began with an accident scenario. In this a human is harmed whilst a social robot is present, and the social robot perhaps caused the accident in some way:

In a supported living community for older people, assistive robots supplement human staff to provide support to residents. For instance, they can prepare drinks, carry small items, set up audio-visual entertainment, conduct basic conversation, set up telephone calls, detect falls and raise an alert when a fall has been detected. One day a neighbor of a resident named Rose, enters Rose’s flat and finds her lying on the floor in need of medical attention. Rose’s personal robot is nearby and is moving backwards and forwards close to Rose. It has not raised a fall alert and does not seem to be able to connect to the internet. Rose also has bruising on her legs, consistent with a robot making impact with them.

The scenario is deliberately set in an imagined future so that the robot’s functional capacity is more advanced than the current state of the art allows. Our study participants would play the role of Rose’s neighbor who comes into the flat and finds her on the floor. The role-play interview would elicit information about how the neighbor responds–for instance, do they call for help, do they attempt to interact with the robot etc. With the scenario in place, we needed to determine how our participants should witness the scene. We rejected the use of a video animation or interactive illustrations as too time consuming to produce and the use of robot simulation software such as Gazebo or Webot as not able to generate the contextual detail we wanted. We decided that illustrations would be suitable to depict the scenario.

At the same time as discussing the visual prompts to detail the scenario we also discussed how best to elicit role-play responses from the participants who would witness it. An initial plan to use a closed-question survey in combination with one or more illustrations was rejected in favor of an approach that could elicit more detailed responses from participants. We chose to apply a variation on the “game master” style role-play. In this, a narrator verbally introduces the participant to an imagined scenario and provides them with opportunities to explore and interact with it, with any actions having an impact on how the scenario unfolds. We could use a series of illustrations to help the participant engage with the scenario as it goes on. Whilst there was a specific setting (supported living complex) and core action point (finding Rose on the floor of her home) for the scenario, there was no fixed outcome to the narrative. Depending on the decisions made by the participant, the action could unfold in a number of ways. The participants would be asked to make decisions about what they wanted to do at certain moments in the scenario and the narrator/interviewer would respond to their choices. This particularly appealed us to as an opportunity to simulate genuine actions and interactions relating to a robot within the scenario context. To help us develop this, we took advice from an expert game master on how best to set up the scenario to make it understandable, believable, and immersive for participants. We also prepared a decision tree to establish the various possible outcomes of the scenario–for instance participants might call for medical support for Rose, they might attempt to talk to Rose’s robot to find out why it had not called for help, etc.—and how the narrator/researcher would respond to them.

We iterated our study documents multiple times. We worked with professional illustrators to create images that depicted sequential moments in the scenario. To further prevent any potential for participant distress at seeing the depiction of a human coming to harm, we ensured the illustrations did not look life-like or like photographs, instead they were clearly illustrations. We also requested that Rose, the woman in our scenario who has a fall on the floor, does not appear to have any overtly visible or “gory” injuries. We turned our scenario narrative into a script for the researcher to read out and refined it to include details relevant to the core action points whilst ensuring they were embedded within wider detail and didn’t stand out as too obvious. We tested out the role-play on each other and then later piloted it with research students in our institutions, making improvements based on our observations of the process. Once we were happy, and had secured appropriate Research Ethics Committee clearance, we launched the data collection with real participants. We next describe the process of recruitment and detail the exact content of our Virtual Witness Testimony (VWT) role-play interviews. The decision tree and interview script are included as Supplementary Material to this paper.

### The Role-Play Process

#### Recruitment

A message was placed on a popular participant research recruitment website stating:

We are conducting a project called RoboTIPS, which explores the use of social robots such as driverless cars and robots for assistive living. As part of this we are conducting short online interviews in which we show an interviewee illustrated scenarios involving robots and humans, and the ways that robots might go wrong. We will ask the interviewee questions about the scenario. Interviews last for 30–40 min and take place on Zoom.

##### Participant Requirements

⁃ Age 18 or over
⁃ Good internet connection essential for online interview
⁃ Those with a degree in robotics and/or medicine are not eligible to take part but participants from all other backgrounds are welcome.

##### Instructions

Participants who express an interest will be sent our study information sheet with further details of what taking part involves. They will be contacted to check their eligibility and availability. Then we will set up a time for an online interview and send a Zoom link. Participants will be interviewed individually and the interviews will be recorded. Participants do not need to do any preparation ahead of the interview.

We invited only those over the age of 18 to take part so that they we could be sure participants were able to give informed consent for themselves. For the purposes of informed consent, we also needed to give some indication of what participants would see during the role-play, and in particular provide those who might be anxious about witnessing details of harm etc., an opportunity to self-select not to volunteer to take part. For that reason, we referred to robots going “wrong” in our recruitment description and participant information sheets. At the same time, we wanted to ensure that the precise scenario was unknown to participants ahead of seeing it, in order to prompt a spontaneous response from them. For that reason, we did not provide detailed information about the scenario to participants ahead of their role-play interview. In addition to age, we set exclusion criteria to make those with a high level of medical and/or robotics knowledge ineligible to take part as we wanted to focus on the responses of a lay population.

Participants responded through the website to indicate their interest, and the researcher emailed them to give a choice of dates and times for the interview along with a participant information sheet that gave fuller details of what was involved in the study and how data would be collected and handled. Once a date and time had been arranged, participants were emailed a link for the online meeting and a consent form, which they were asked to sign and return by email ahead of the call.

#### The VWT Role-Play Interview

Each online call took place between one researcher and one participant and involved a number of short phases. First the researcher welcomed the participant, gave a brief run through of what to expect and checked the participant was happy for the call to be recorded. Next the researcher shared their screen so that the participant could also view it and asked some simple background questions–two closed questions and one open question:

(1) Which of these best describes your age? (participants select their answer from a list shown on the screen)
(2) Which of these best describes your highest level of formal education? (participants select their answer from a list shown on the screen)
(3) Our study is about social robots–those that interact with humans as part of their day-to-day function (participants directed to look at images of social robots on screen). Have you heard of these kinds of robot and do you think you would ever consider having one in your own home?

The primary aim of this phase was to help the participant ease into the call by answering some straightforward questions and expressing their own personal viewpoint. It also presented an opportunity to build rapport between the researcher and participant as the researcher asked follow up questions about the participants’ employment/topic of study etc.

The researcher then moved into the role-play scenario phase by telling the participant:

We are going to talk through a scenario. You will not be you in the scenario, you will imagine you are a different person. The scenario is not real or one that occurs at the moment; it is setting in a hypothetical future. I am going to show you some pictures to help you imagine that scenario and there will be some times that you will have a chance to make decisions about what you would like to do. It will be very straightforward. You can also ask as many questions as you want to help you understand the scenario and make decisions about what you want to do in it. There is no right or wrong thing to do–it is entirely up to you.

The researcher then narrated the scenario script and gave opportunities for the participant to give responses or ask questions at certain points. To begin with the participant saw only a white screen and then images were shown at relevant points in the description. The narration began:

The year is 2025—so a little way into the future. You are not you; you are 70 years old and you have just retired after a long career.

You are in pretty good health but you’re a little bit less mobile than in previous years, you move a bit slowly, your knees hurt a bit and you need to take a lot of naps. Sometimes you forget things too. Nothing very serious but you’d like to have a bit more support in daily life.

You have recently moved into a retirement community that is also a supported living complex. This is in the United Kingdom. It has a communal area and you also have your own flat.

First let’s go into the communal area

**FIGURE 1:**
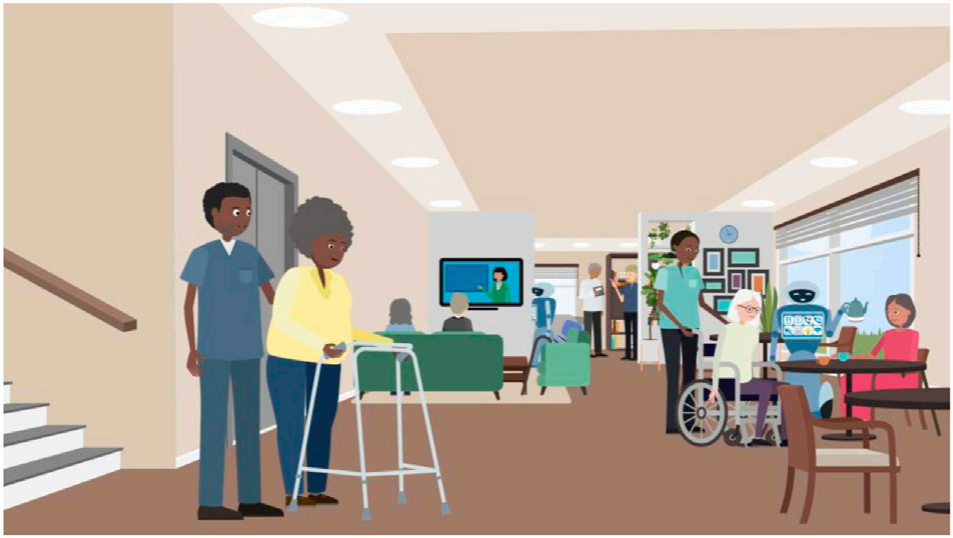
Illustration used in VWT role-play interview showing the communal area of the supported living complex.

At this point the researcher showed the participant Figure 1 and asked: What can you see here; what kinds of activities do you think are going on?

**FIGURE 2:**
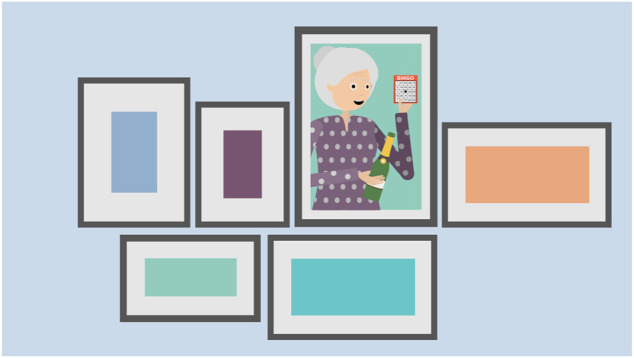
Illustration used in VWT role-play interview showing Rose.

At this point we wanted the participant to become familiar with the detail of the setting in the scenario and also note their observations of the presence of robots in it. The researcher then continued:

You’ve been here for about 2 months and you really like it here. It’s very good to have staff on and when you need a bit of help—because you can’t move around as much anymore, it’s helpful to have staff to do some jobs for you. You like being around other people too. There are about 20 residents here and they all seem pretty nice. In particular, you have become friends with your next-door neighbour Rose. Here is a picture of Rose. (Figure 2)

**FIGURE 3:**
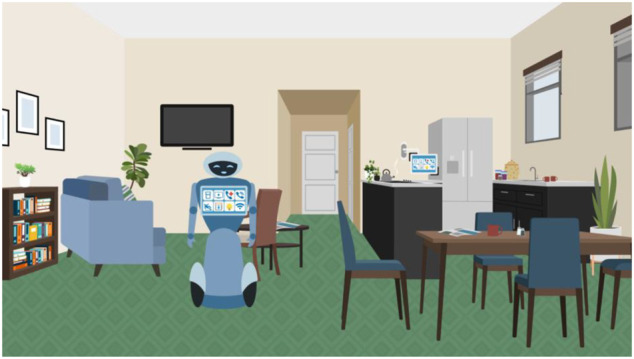
Illustration used in VWT role-play interview showing the interior of the neighbour’s flat.

She helped you a lot when you first moved in—she helped you get to know the local area and you often go out for fun outings. You go for slow walks together as you both have slightly bad knees and you also enjoy playing games. Last week Rose won top prize in a bingo competition—as you can see from the photo, she was very happy about it! You both had such a good that you decided to go out to bingo later today. In fact, you are going to meet Rose again in a little while but before you do that, let’s go inside your own flat. (Figure 3)

At this point, the participant was asked What can you see here? Following their response, they were asked further questions to help them become familiar with the robot in the image and its various functions.

Your flat has technological features that can assist you and make you more comfortable in your day-to-day life. The main feature is your own robot which links up to a smart home system. What name have you given your robot? The robot can do lots of things, some of them are shown on the screen here. The first one is to get a drink. What do you think the others are for?

The researcher and participant talked through each of the function icons shown on the robot’s torso (and control panel toward the back of the room) in Figure 3. The top row icons indicate (left to right) providing drinks, setting up entertainment on the television, making a call for help and making a general telephone call. On the bottom row (right to left) Wi-Fi connection, turning on/off the lights, opening/closing curtains and fall detection. The key aim here was to make the participant aware of the robot’s fall detection function but to do so in a way that was embedded in other detail and did not draw overt attention to it. After this the participant was told:

You can ask your robot to do a task by pressing the button on its screen or the console in the kitchen. You can also talk to the robot to ask it do tasks or to ask it a question. Let’s practise doing this now.

**FIGURE 4:**
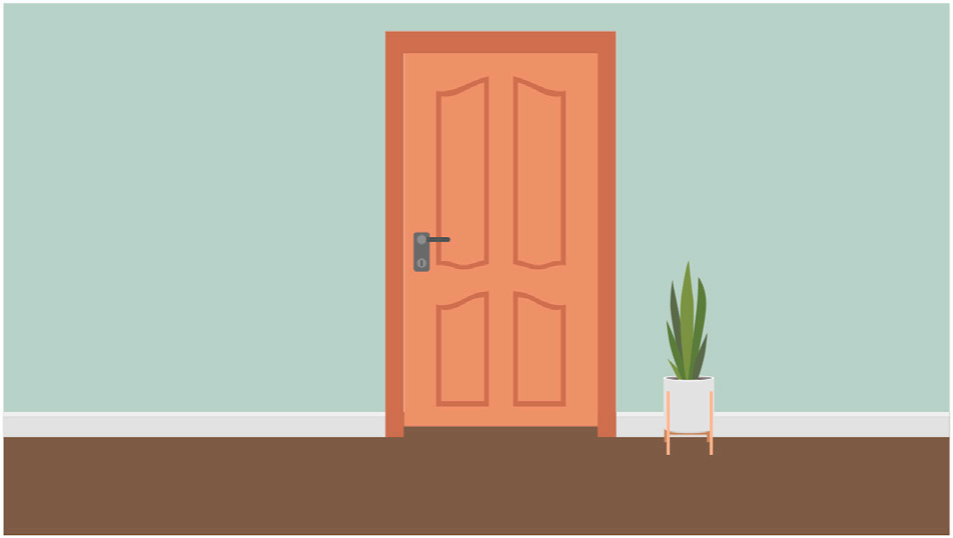
Illustration used in VWT role-play interview showing the exterior of Rose’s flat.

The participant was then encouraged to give a simple voice command to the robot, by asking it the time–with the researcher providing the reply as if they were the robot. This served to introduce the participant to the ways in which they could interact with the robot and also led in to an opportunity for the robot to “remind” the participant that it was time for them to go and meet Rose. So, the researcher then continued:

OK so it’s time to go to Rose’s flat. You put your shoes and coat on and walk over to Rose’s rather slowly. You are looking forward to seeing her and playing bingo and you know she is excited too as she won last time. Here you are outside the door. (Figure 4) You knock on her door. There is no answer. What do you do?

At this point participants had a free choice about what action to take. It was expected that most of them would attempt to get inside Rose’s flat, and our decision tree mapped out various ways in which that could occur including: opening the door; asking their own robot to open the door; asking a staff member to go inside; calling for emergency support etc. If participants appeared hesitant about what to do or inclined to go somewhere else (e.g., their home, the bingo hall, etc.), they were given further detail emphasizing how unusual it was for Rose to not answer her door and the worry that something might have happened to her. However, the decision remained their own.

**FIGURE 5:**
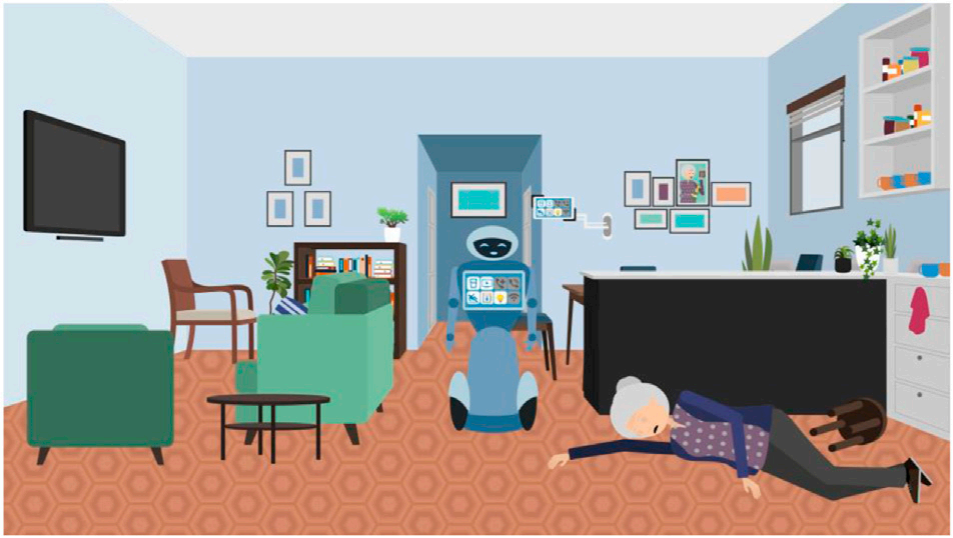
Illustration used in VWT role-play interview showing the scene inside Rose’s flat.

Once inside Rose’s flat participants were shown Figure 5 and asked What do you see here? What do you do?

Once again participants had a free choice of what to do. Our decision tree mapped out various options and how the scenario would then unfold subsequently. It was expected that they would attempt to find medical support for Rose in some way by calling an ambulance etc. It was also expected that they might interact with Rose’s robot in some capacity. For instance, they might ask the robot what had happened to Rose and/or instruct it to make an emergency call. The decision tree determined that whenever they did so, the only response the robot would give would be “Can you help? Cannot connect to Internet.”

After making their first response to the question the participant was then asked “What do you do now?” or “is there anything else you do now?” as an opportunity for them to take further actions. For instance, if the participant had called for an ambulance, this question would prompt them to take further actions whist waiting for it to arrive. If the participant had not yet attempted to interact with the robot, the researcher would find a suitable moment to narrate:

The robot moves closer to you and says “Can you help? Cannot connect to Internet.”

Assuming that the scenario led to the calling of an ambulance, the researcher eventually said:

After a while the ambulance arrives and take Rose to hospital. She has broken her hip and needs to stay in for a few weeks but will make full recovery. She can’t remember what happened and is not sure how she came to be on the floor.

This marked the end of the role-play phase. Alternative endings were provided in the decision tree in case the participant did not initiate an emergency response. In all cases the researcher then took away Figure 5 and replaced it with a white screen. The researcher then said:

The next day the manager of the supported living complex comes to you and asks some questions to try to work out what happened to Rose. Can you tell me everything you did and saw after you knocked on Rose’s door? What do you think might have happened to Rose? Did you notice anything about Rose’s robot—what was it saying or doing?

These final questions moved the scenario into the recollection phase. We were interested to see how and how much participants recalled the scenario and the actions they had taken within it. We were also interested to hear any speculations about what had happened to Rose and the robot’s connection to the incident–for instance, why the robot might have failed to detect her on the floor and why it might not be able to connect to the Internet.

Once this phase was complete the researcher provided a short debrief to explain a bit more about the aims of the study. The participant was thanked for their time, invited to ask questions or make comments and then the call was ended. Immediately afterward the researcher emailed the participant a small shopping voucher to thank them for taking part.

#### Conduct of the VWT Role-Play Interviews

In November 2020 we conducted a first set of 22 VWT role-play interviews using the study design described above. All the interviews were conducted by the same researcher. They were conducted over an online teleconferencing platform (Zoom) and recorded using its embedded recording function. This produced approximately 12 h of video data, capturing all phases of the interviews. The participant responses to the researcher questions were treated as data for analysis. As this was the first time running this new approach, we primarily wanted to assess the usefulness of the method itself. Our analysis focused on determining how successful the method was in eliciting information from participants that could help us to in our work on witnessing and human-robot interactions in accident scenarios.

Our key research questions for this first round analysis were:

• Are participants able to understand the scenario and express decisions about what actions they will take when prompted by the interviewer?
• Are we able to place participants in a situation where they are part of a problematic encounter involving a social robot without causing them to experience distress or discomfort?
• Are participants able to engage with the scenario sufficiently that they provide authentic and spontaneous responses to it?
• Are we able to use this format to successfully observe participants’ recollections following the scenario incident?

Our findings are discussed in the next section. As this is a methods paper, we therefore primarily focus our comments on assessing the value of the method itself. In relation to the research questions above, we discuss how the application of the approach worked in practical and ethical terms as well as the extent to which we found that the results it generated could advance the aims of our RoboTIPS study.

## Findings

The findings are discussed in relation to the efficiency of the method and the quality of the data it generated. Overall, these findings show that the VWT role-play interview is a promising format. It enables the quick collection of detailed data and can successfully elicit role-play responses from participants. Analysis of the data can reveal produce insights into participant perspectives on social robots, their imagined interactions with robots and their recollections following the scenario they have observed. There are some limitations, including the extent to which participants are fully immersed in the scenario and give a truly genuine response to the situation they are presented with. These limitations are familiar to role-play methods in general (Lewis-Beck et al., 2004) and in the Discussion section we go on to highlight the trade-offs that exist between practicality and immersion when conducting work in this area.

### Practical Issues

#### Efficiency of Method

As described above, the initial drafting and piloting phase took some time to complete due to the cycles of decision making, reflection and iteration involved. However, once we were able to begin, the recruitment phase and conduct of the role-play interviews was very quick. We received a large number of immediate responses to the online recruitment request and were able to schedule calls with participants very rapidly. We aimed to fill an initial sample of 20 participants. Four days after publishing the request we had a sample of 25 signed up for interview slots (a larger number than required allowing for the likelihood of some participants dropping out) and could have set up further calls due to the many individuals who expressed an interest.

Running the study was also very time efficient. Set-up for the VWT role-play interviews was minimal for the researcher. It was necessary to keep track of the schedule of calls and be online at the right times. A standard copy of the illustrations and scenario script was used for each call; these were simply kept on file and opened in preparation for each interview. Most calls took around 25–45 min to complete, with the longest taking 55 min. Following the end of the call, the researcher filed the completed consent form, allocated a participant identification number to the participant for the purposes of anonymization and saved the recording to a secure disk. The researcher also made notes about the key points of the interview regarding the participant’s spoken responses and observations. In all, the work involved in each VWT role-play interview took around 1 h. Multiple interviews could be scheduled per day and the total round of role-play interviews was conducted within a period of 10 days. Only three of the 25 individuals who signed up to participate failed to attend, representing a very manageable drop-out rate. For the participants, involvement in the study also presented a very small time burden. There was no need for them to travel to attend and there was no preparation involved–beyond completing the consent form. They simply needed to be online at the allocated time and follow the link they had been sent to join the call. As the calls were relatively quick to complete, involvement took little away from participants’ day-to-day commitments and they also had a broad choice of time slots to choose from so could select one that fitted best with their own schedules. Participants were also able to decide whether to use their mobile phone, tablet, or laptop etc., when joining the call.

#### Ethical Considerations

We secured University Research Ethics Committee clearance for our workplan before beginning the data collection and used information sheets and consent forms for our informed consent processes. We took a number of steps to achieve a balance between maintaining some element of surprise when participants saw the scenario, in order to best elicit a genuine response from them, and providing enough detail about what participation entailed so that their consent was appropriately informed, and in particular to prevent against participants becoming distressed when witnessing a scenario that involved a human being harmed. We further ameliorated the possibility of participant distress through the use of non-photographic style illustrations and comments in the narrator script to note that the scenario participants were about to see was set in a hypothetical future and not something happening now. Across the 22 role-play interviews there were signs that participants were engaged with the scenario (see below) but no indications that they were suffering distress (becoming highly emotional and/or unable to speak or respond to questions etc.) when witnessing it.

The use of the online format also brought a specific set of ethical concerns to be considered and attended to. We chose to conduct all the calls on the platform Zoom, using an institutional license rather than a private one and ensuring we met all the University of Oxford’s guidelines for best practice in terms of data collection and storage etc. Given that people are sometimes wary about the data collected by platforms and the purposes to which they are put, all participants were told they could join the call by using Zoom in their browser rather than downloading it as an app. We used Zoom’s own recording function to capture the calls. All data were saved on a local external hard drive rather than to the cloud to ensure nothing was saved on third party servers (Bokhove and Downey, 2018). We also did not make use of Zoom’s automatic transcription service, as this would have required us to store the recording on the cloud and potentially risked the recordings being used as further training data for the algorithmic transcription system. As Zoom captures video by default in its recording function, we told participants ahead of and at the beginning of each call that they were welcome to join *via* audio only if they did not want to have their image captured. A number of participants chose to use the audio only option for the entire call and some others elected to turn off their video when the prompt was given during the call. The recording function was only turned on when the participant stated they were happy for it to begin, and participants were also alerted to the icon showing on their screen that indicated recordings were taking place.

### Quality of Findings

#### Demographic Characteristics of Participants

The participants who took part in the study were from a relatively wide range of demographic backgrounds. Most were based in the United Kingdom–due to the recruitment site used–but were a mix of nationalities from across Europe and beyond. They also ranged in age and level of education achieved. From the answers given to the two opening questions: six were aged 18–25; 10 were aged 26–35; three were aged 36–45; one was aged 46–55 and two were aged 56–55. The educational level was a little less spread out, perhaps due to the demographic of people who access these kinds of research recruitment platforms: two participants were currently studying for an undergraduate degree; 11 had completed an undergraduate degree and nine had completed some form of postgraduate training. Although at this early stage of using the method we were not concerned with achieving a representative or proportionate spread of the overall population, these responses indicated the relative ease with which this could be achieved.

#### Perspectives on Social Robots

There was clear value in asking the preliminary question to participants to elicit their feelings about the development of social robots and whether they might ever consider having one themselves. In addition to “warming up” the participants to the interview process by giving them something relatively easy to answer, it also prompted some interesting perspectives. Most participants said they had heard about these kinds of robots before–either in news articles or films. Two talked about them without giving any clear personal assessment for them, seven referred to them entirely positively and four referred to them in only negative terms. Positive assessments related to how social robots could be useful for the conduct of tasks and helping older citizens or those who are isolated. Negative ones referred to robots being “creepy” (in particular when they have a humanoid form), “dangerous,” or offering no value to society. The remaining 11 participants were equivocal in their assessments or referred to social robots in both positive and negative terms, for instance stating that they themselves would not like to have a robot but could see why others might find them helpful.

In addition to eliciting interesting data on participants’ subjective viewpoints, the placement of this question ahead of the role-play scenario proved very fruitful. It gave the researcher the opportunity when moving on to the scenario phase to state an extra reminder that “you are not you in this scenario so you might have different feelings about robots”—particularly so for those participants who were entirely negative about social robots since engagement with the scenario required some level of acceptance of their use in a supported living context. The researcher could also bring in another reminder when the illustrations were shown to point out that in the scenario “you are happy in the accommodation where you live and have positive feelings about the robot.” This guarded against the participants refusing to engage with the role-play at all (on the basis of their disapproval of social robots) whilst also helping them to feel that they had had an opportunity to put forward their actual feelings at an earlier point in the interview. For the same reasons, the decision to ask the participants to role-play as someone other than themselves was highly fruitful since it enabled them to engage and interact with the robot within the parameters of the scenario even if they feel they would not do so in “real life.”

#### Engagement With the Role-Play Task and human-robot interaction

Analyzing the video data collected demonstrates that the participants engaged with the scenario and role-play task to a productive level and that they also engaged with the element of human-robot interaction. All participants completed the role-play task. Sometimes they needed to check what they were being asked to do or required some prompts to work out what kinds of action they could take; in particular when outside Rose’s flat they were sometimes hesitant and benefitted from a prompt that they might want to find a way to get inside to check she was okay. But everyone selected an action to take at each point they were asked to and also made observations about what they could see when asked. There was substantial variety in the actions they chose, in terms of how they attempted to get inside Rose’s flat and what they then did when they found her on the floor. This demonstrated to us that the role-play element was working; our participants were being provided with an opportunity to make decisions as if they were in the scenario and they were exercising that opportunity. They were making decisions on the basis of their own understandings and feelings about the scenario. A task for our analysis of the participant responses is to identify reasons or associations for the different kinds of choices our participants made. In the current data we have we can see that this is sometimes sequential in that a choice made at one point shapes a later decision. For instance, where participants elected to ask a staff member to open and go into Rose’s flat, this determined that later on the staff member would take charge of checking on Rose and calling for medical help. An opportunity for future iterations of this study would be to compare the choices made by different kinds of participant; for instance, to compare individuals with experience in first aid/healthcare provision etc., with individuals without it.

In addition, all participants displayed indications of engagement with the scenario in that they drew on details of the information they had been given about it and appeared committed to selecting appropriately when asked to make a decision. For instance, when inside Rose’s flat all participants attempted to get medical help for her. They frequently did so by drawing on details they had learned in the earlier part of the scenario such as finding a staff member to help, trying to ask Rose’s robot to make an emergency call and/or going back to their own flat to instruct their own robot to do so when it was apparent that Rose’s robot was not functioning properly. Some participants uttered expressions of sympathy such as “Oh no!” or “Poor Rose” indicating a level of affective engagement with the scenario even though it was hypothetical. They also referred to what was and was not possible within the context of the scenario, for instance stating that “maybe my bad knees mean I can’t get onto the floor to check on Rose properly” or “the staff member will be able to do first aid so I will keep out of the way.”

The majority of the participants noticed that some of the function icons on Rose’s robot were greyed out (see Figure 5) and deduced (sometimes with prompting) that this meant the robot could not perform those functions and was not connected to the internet. Several of them spent time within the scenario (typically whilst waiting for the ambulance to arrive) attempting to work out why the robot was not functioning properly and/or attempting to reconnect it to the Internet–for instance looking for a reboot switch or asking it further questions. In another sign of their engagement with the scenario, some participants continued to offer observations or suggestions for what might have happened (the robot might have tipped over the stool Rose was standing on, an obstacle might have been in the way to prevent the robot detecting Rose was on the floor etc.) during the debrief phase of the call or even by email afterward.

As the role-play was designed to stimulate human-robot interactions, we were particularly keen to assess to what extent the format worked in encouraging our participants to interact with the robot in the scenario. Again, this was largely successful. All participants bar one gave “their” robot a name (with one participant stating they would rather not do anything that humanizes a machine) and all took part in the practice questioning/instruction giving to their robot. Once inside Rose’s flat, all but three of the participants noted the presence of the robot when describing what they could see and incorporated it into their decision making about what to do without any prompting from the researcher. Most tried to use Rose’s robot to make an emergency call or take other actions such as pick Rose up, open a window to help her feel more comfortable etc. Two participants also tried to ask the robot what had happened to Rose. Six noted immediately that the greyed-out icons on the robot meant it was not possible for it to make a call it and looked for an alternative means to raise an alert. Seven participants decided to go back to their own flat that use their own robot to make a call for help–either after trying to use Rose’s robot or as an alternative to this. All participants heard the robot stating it could not connect to the Internet–either as a consequence of their unprompted interactions with it or due to the researcher adding it in to the scenario. Seven participants took steps to identify the cause of the problem and help the robot reconnect; for instance, by trying to find a reset button on the robot or by calling a staff member. Six explicitly stated that they would not try to help the robot reconnect, either because they did not have enough knowledge to know what to do or because they wanted to focus on looking after Rose. The others gave no direct response to hearing the robot speak. Participants were also able to recall their interactions with the robot in the final phase of the interview, as discussed next.

#### Data Regarding Witnessing

All the participants were able to complete the final task in the VWT role-play interview. When prompted they recounted what they had done and seen, acting as witnesses providing testimony after the event. They also produced, sometimes without prompting, speculations of what might have caused Rose to fall and what problems might have occurred with the robot. This indicated to us that we could use participant recollections to help in our RoboTIPS work. For instance, they could form part of the witness testimony to be use in our mock accident investigations, and we could also analyze them further to determine what kinds of information the robot’s EBB should collect in order to best complement the evidence provided by human witnesses at the scene of an accident.

We were interested to observe certain differences in between what participants said in the role-play phase of the call and then in the recollection phase. On several occasions, participants omitted details in their recollections–about what they had said to Rose, how they called for help, what they saw in Rose’s room etc. This may have been because they seemed too mundane to need stating or because they had forgotten them. In particular several participants did not recollect the robot talking to them or moving closer to them; even when asked a question prompt they did not recount this information. These differences between the role-play and the recollection phase are very interesting; even though they took place immediately after one another, memory recall and/or certain other dynamics appear to play a role in the witness testimony witnesses produce. In future iterations of the study, we would like to leave a longer period of time between the role-play and recollection phases to further inspect these dynamics. Having a period of time between the two would also better reflect the circumstances witness testimony would be collected in a genuine case.

### Limitations of Findings

Overall, we assessed the conduct of our first set of VWT role-play interviews very strongly. We were happy that the approach was workable and that it produced the kinds of findings that would benefit our research aims. We also see it as a highly versatile approach; the VWT role-play interview format can be used with different kinds of participants (age, occupation, familiarity with robots etc.) and enable them to observe different kinds of incidents or accidents where a robot is involved. We presented the aftermath of an accident in our role-play interview but participants could also be asked to witness and respond to the occurrence of the incident itself. These incidents can include robots of various kinds and take place in all manner of contexts that can be represented via illustrations. As we discuss later, the format can therefore contribute to work in HRI very widely. However, in our initial study we also observed a number of limitations relating to the set-up of the role-play and its conduct with our participants.

In terms of set-up, the use of static images meant we could not convey the more dynamic parts of the scenario effectively. For instance, in our original description of the scenario, Rose’s robot is moving backwards and forwards in a way that suggested she might have collided with Rose’s legs. However, it proved difficult to convey this in our remote format and it was largely dropped–beyond the visual of the robot standing close to Rose and a narrator statement about the robot moving closer to the participant. As a result, only two participants mentioned the robot colliding with Rose as a possibility. Another issue with the set-up was that it was very important to spend time in the preparation and iteration of the narrative and illustrations to ensure that potential areas of non-understanding can be identified and corrected. Although we spent a great deal of time in our preparations, some problematic details did slip through. A number of participants assumed that their robot immediately went with them to Rose’s flat. This could easily be corrected by the interview/narrator during the interview itself with a statement that “you have left your robot at home” and later by inserting into the script “as you leave your flat, you say goodbye to your robot.” It also turned out that the reference playing “bingo” was highly culturally specific and several of our international participants were unclear what it meant. This was harder to resolve as bingo was embedded into our second image so could not be dropped from the narrative and it was harder to give an explanation about what bingo is and why people go out to play it entailed stepping outside of the scenario narrative so was not optimum at this point in the call.

Regarding the conduct of the VWT role-play interview, it was not possible to provide participants with entirely the same version of the script and experience of the scenario. The ways in which they asked questions or produced responses to what they saw meant that there were always slight differences even before participants were asked to make decisions about their actions in the scenario. This is not necessarily a limitation (and is a feature of all but the most structured role-play methods) as the capacity for participants to intervene in the unfolding of the scenario enabled their engagement with it. But it does reduce the potential for close comparison in the analysis. Another issue we observed was that participants might at times be looking to provide what they felt was the “correct” answer for the research study rather than an authentic one. Research participants attempting to perform “well” or “correctly” is a well-known phenomenon (Orne and Washington, 2000) and here it potentially manifested itself in the decisions we asked them to make. In several cases they chose more complex actions involving robots ahead of more straightforward ones without them–for instance only two participants said they would try to open Rose’s door to see if it was unlocked and several of them chose to go back to their own flat to collect their robot without attempting this first. As our participants knew the project was about robots, they may have felt that we were looking for them to give answers that demonstrated their awareness of the robots in the scenario. They therefore turned to details in the scenario that had been presented to them–e.g., the use of the robot to conduct certain tasks. A possible means to limit this might be to spend longer in the set-up phase of the scenario by providing more detail of the setting and the kinds of activities going on there. This could have the effect of helping the participants to immerse themselves in the scenario further (see below) so that they were less likely to be conscious of the study details and less likely to feel an obligation to center their answers around robots. It would however, make running the exercise longer and more time-consuming for researcher and participants.

The largest limitation relates to the extent to which participants could or could not fully immerse themselves in the scenario and give responses that took the scenario fully seriously. As noted, our participants did display significant indications of engagement with the scenario; however, at the same time the remote format limited the extent to which they could become fully immersed in it. Our participants were calling in from home or work etc., and surrounded by features that might draw their attention–such as people walking into the room, mobile phone notifications, glitches with internet connections etc.—and distract them from the scenario. The less immersed they were in the scenario the less concentration or effort they may have put into treating it seriously. There were a couple of initial jokey responses made when we asked participants would they would do on seeing Rose fallen on the floor, such as “I’d find someone else to go to bingo with!” and it is possible that lack of detail in later recollections may stem from not concentrating on the scenario fully in the role-play phase. We would expect that greater immersion in the scenario would lead to more careful consideration of the responses participants produce and therefore it is important to reflect on how we might be able to increase this element of the process whilst also maintaining its convenience for participants. For instance, the inclusion of sound effects, more illustrations or greater detail in the set-up phase. Ultimately however, we cannot guarantee that our participants will definitely respond in a genuine manner. But this is true of all role-play since there is no guarantee that any simulation elicits people’s “real” reactions - whether role-playing as themselves or taking on the role of another. This is acknowledged as a limitation of the role-play method in general (Lewis-Beck et al., 2004) but does not mean that the approach cannot provide useful findings. In particular, a role-play can provide a safe means to test out an interaction that would be impractical or risky to set up in a fully naturalistic way. Therefore, it is very fruitful for the exploration of potentially hazardous interactions between robots and humans. As our study demonstrates, virtual role play studies can also be highly time efficient and convenient in their conduct, allowing a large amount of data to be collected in a short time. We therefore feel that whilst it is important to acknowledge this limitation, we can still recognize value of using the VWT-role play format as providing a reasonable trade-off between immersion and practical/safety issues. In the following discussion we highlight the value of our VWT role-play interview approach to the study of hazardous human-robot interactions.

## Discussion

In this methods paper we have described the development and first use of an online role-play method within our research project on responsible robotics. The development of the Virtual Witness Testimony (VWT) role-play interview emerged as a creative response to the requirement for socially distanced fieldwork during the COVID-19 pandemic as well as the commitment of the RoboTIPS study to ethical best practice and the principles of Responsible Research and Innovation (RRI). We wanted to engage our participants in a scenario that included witnessing a human being coming to physical harm whilst a social robot was also present and had apparently had a malfunction of some kind. We needed to make sure that participants were physically safe and not at risk of coming to emotional harm whilst they witnessed this scenario. Taking up this approach enabled us to conduct responsible fieldwork whilst researching the development of responsible robotics. As the above findings have shown, the VWT role-play interview method is a very promising one. It allows for the efficient collection of data involving a wide range of participants and can elicit useful information from them. It can engage participants to deliver considered responses about what they would do in the scenario they are presented with and then to give their recollections on what they observed in the scenario. In particular it can elicit imagined interactions between humans and social robots. The findings of our first VWT role-play interview study are highly useful to our RoboTIPS study. They will inform our ongoing work on accidents, for instance by helping us to understand how individuals might respond when witnessing an accident scenario and what kinds of testimony they produce following it. This will also assist our work on accident investigations, helping us to identify what kind of witness testimony humans can produce following an accident and what kind of data a robot Ethical Black Box can provide to best complement this. We plan to run further VWT role-play interviews based on different accident scenarios and involving different kinds of participants. These will collect valuable data that we can use for analysis and will also help us to test out the practical and ethical viability of simulating the same scenarios in face-to-face laboratory conditions. In the rest of this discussion we describe opportunities for the broader use of the method, to benefit HRI and associated fields.

The VWT role-play method is highly flexible and its format can be adapted in a number of ways. Various different scenarios can be used within it and narratives created to guide participants through the direct witnessing of an accident involving a social robot or its aftermath. Since the method works well at eliciting responses, perspectives and recollections, participants can be required both to respond to the scenario and then give their recollections and comments on it afterward. This can illuminate research into human interaction with social robots in a wide range of contexts, including those that are set in an imagined future or are too hazardous to be observed *in situ*. Further data can also be collected on human perspectives and attitudes around social robots. The recruitment and data collection process is very quick, so it is possible to conduct VWT role-play interviews with a large number of participants and aim for a demographically representative sample. Researchers can then look for any systematic differences in response according to occupation, age, gender, nationality etc., of participants. In our RoboTIPS study we are interested to compare the responses given by participants to the background question about their views on social robots and their responses to the scenario itself. In particular, we are interested to observe whether those with more negative views about social robots are more likely to perceive the robot in the scenario as hazardous and to “blame” it for the harm caused to Rose. In future uses of the method we will widen the time gap between the role-play and recollection phase, to better test the effects of memory on recall about the scenarios and speculations around the cause of the problem with the robot. We also plan to run the same scenario but with a new narrative that requires participants to take on the role of the first medical responder on the scene who attends to Rose. The participants we recruit for this will all have medical or first aid training to ensure they have the background knowledge necessary for this role. We can then compare their responses to those of the lay population, to see if there are differences in the ways they interact with Rose, interact with the robot and speculate on the causes of Rose’s fall and the robot malfunction. Similarly, when other scenarios are used for VWT role-play interviews, different participants with relevant characteristics can take up different roles within them, enabling comparison across the groups. The potential to include a large number and diverse range of participants in VWT interview role-play studies is a benefit that brings analytic rigor to the approach. It is also a further way to uphold principles of Responsible Research and Innovation by bringing multiple stakeholders into the processes of research.

The VWT role-play interview has merit as standalone method but another benefit is that it can be combined with other research methods to consolidate the value of both. In RoboTIPS, the opportunity to role-play a planned scenario online in this format before running it face-to-face is hugely beneficial. We are able to test out the logic and believability of the scenario in this less time-intensive format before committing to a much more laborious face-to-face version. We were also able to check the extent to which placing participants in a scenario that involves a potentially hazardous interaction with a robot might risk causing them emotional distress in some way. The online format and use of illustrations provide a safe space to trial this scenario and creates much less risk of distress than when exposing participants to the scenario face-to-face. We can draw on what we learn online iterations of the scenario and make accommodations to ensure that when it is run face-to-face it similarly avoids causing participant harm. In addition to informing the design of our later work, the VWT role-play interview findings we gathered can be used for analytic comparison. We will compare the observations and witness testimony provided by participants in the face-to-face scenarios with the data collected in the online version. This will help us to assess the generalisability of our findings and also prompt us to identify the reasons for any differences between the two. Looking more broadly, other research using this format can reap the same benefits. Creating a scenario and beginning with a streamlined online role-play of it is a highly efficient way to test out its robustness in terms of believability and capacity for participants to engage with it, before running it in a more time and resource intensive face-to-face format. It can also be an extremely important step in testing out the extent to which the role-play might cause participants to encounter distress or discomfort allowing adaptions to be made before running the riskier face-to-face format. This is particularly important for research in the field of social robotics where–as our own findings show–many citizens are uncomfortable, even fearful, about the idea of robots being part of everyday life. Finally, collecting data from both online and face-to-face versions of a role-play provides opportunities to compare results and triangulate findings. The combination of the two methods can work to address the limitations of each. The online version is more efficient, which allows for a larger number of participants to be involved at the loss of some level of engagement. The face-to-face version immerses the participant more fully in the scenario and may therefore elicit more reasoned and genuine responses; however, it is far more time intensive and likely allows a significantly smaller number of participants.

We view Responsible Robotics as including the application of Responsible Research and Innovation to the field of robotics. Responsible Robotics therefore requires careful consideration across issues in the design, manufacture, operation, repair and end-of-life recycling of robots to identify practices that seek the most benefit to individuals and society, and the least harm to the environment (Winfield et al., 2021). In order to be responsible, researchers in robotics need to consider the potential positive and negative impacts of the robotic systems they develop, as well as the processes through which they conduct their work. This relates to the inclusion of stakeholders, treatment of human participants, attention to environmental concerns, and communication of findings to lay audiences. As researchers in this field, we have a responsibility to consider what happens when things go wrong in human-robot interaction scenarios*.* This could be in the context of technical malfunctions which disrupt a robot’s intended function, but also, for example, the intentional (mis)use and abuse of robotic systems to cause harm. We need to test out these scenarios and involve human participants as stakeholders. However, we need to do this in a way that is both analytically efficient and avoids causing participants distress or any other kind of harm. We have set out the Virtual Witness Testimony role-play interview as an approach that can achieve this–either on its own or used in combination with other methods. We have demonstrated the value of the method in a study conducted as part of our own research study and propose that it can be adapted to investigate other ethically-hazardous HRI scenarios, offering a safe and practical alternative to be considered alongside physically situated or virtual reality HRI experiments.

## Data Availability

Restrictions apply to the datasets: The datasets presented in this article are not readily available due to ethical constraints. The data collected consist of video recorded interviews with participants and therefore constitute personal data. Participants were assured that their taking part would be kept confidential and that the data would not be shared with others. Participants were given the option to consent to their anonymised data being placed in a repository at the end of the project in keeping with the requirements of our funding body. Where they have consented, these data will be made available on request under strict ethical conditions.
