# Supplementary material for: Role-Play as Responsible Robotics: The Virtual Witness Testimony Role-Play Interview for Investigating Hazardous Human-Robot Interactions
Source: Front Robot AI. 2021 Jun 29;8:644336. doi: 10.3389/frobt.2021.644336 (PMC8275931; doi:10.3389/frobt.2021.644336)
Supplement: Supplementary file 1 [file DataSheet2.PDF]

Role-play as Responsible Robotics: The Virtual Witness Testimony role-play interview for investigating hazardous human-robot interactions

Appendix One: Researcher script for VWT role play interview

Introduction

Which of these best describes your age? (participants select their answer from a list shown on the screen)

Which of these best describes your highest level of formal education? (participants select their answer from a list shown on the screen)

Our study is about social robots – those that interact with humans as part of their day-to-day function. (participants shown images of social robots on screen). *Have you heard of these kinds of robot and do you think you would ever consider having one in your own home?*

Role-play

We are going to talk through a scenario. You will not be you in the scenario, you will imagine you are a different person. The scenario is not real or one that occurs at the moment; it is setting in a hypothetical future. I am going to show you some pictures to help you imagine that scenario and there will be some times that you will have a chance to make decisions about what you would like to do. It will be very straightforward. You can also ask as many questions as you want to help you understand the scenario and make decisions about what you want to do in it. There is no right or wrong thing to do – it is entirely up to you.

That will take about 15 minutes and then at the end I will ask some questions about it.

The year is 2025 – so a little way into the future. You are not you; you are 70 years old and you have just retired after a long career.

You are in pretty good health but you're a little bit less mobile than in previous years, you move a bit slowly, your knees hurt a bit and you need to take a lot of naps. Sometimes you forget things too. Nothing very serious but you'd like to have a bit more support in daily life.

You have recently moved into a retirement community that is also a supported living complex. This is in the UK. It has a communal area and you also have your own flat.

First let's go into the communal area (participant shown Image 1). *What can you see here; what kinds of activities do you think are going on?*

You have your own flat but in the wider complex there are also staff around – a caretaker to do physical jobs and care staff who can do first aid or provide help in other ways. There are also robots here too. They can provide drinks and conversation and do other things too. We will find out more about them later.

You've been here for about 2 months and you really like it here. It's very good to have staff on and when you need a bit of help – because you can't move around as much anymore, it's helpful to have staff to do some jobs for you. You like being around other people too. There are about 20 residents here and they all seem pretty nice. In particular, you have become friends with your next-door neighbour Rose. Here is a picture of Rose (participant shown Image 2).

She helped you a lot when you first moved in – she helped you get to know the local area and you often go out for fun outings. You go for slow walks together as you both have slightly bad knees and you also enjoy playing games. Last week Rose won top prize in a bingo competition – as you can see from the photo, she was very happy about it! You both had such a good that you decided to go out to bingo later today. In fact, you are going to meet Rose again in a little while but before you do that, let's go inside your own flat (participant shown Image 3).

What can you see here?

Your flat has technological features that can assist you and make you more comfortable in your day-to-day life. The main feature is your own robot which links up to a smart home system. What name have you given your robot? *The robot can do lots of things, some of them are shown on the screen here. The first one is to get a drink.* What do you think the others are for?

You can ask your robot to do a task by pressing the button on its screen or the console in the kitchen. You can also talk to the robot to ask it do tasks or to ask it a question. Let's practise doing this now. You've forgotten what time you are meeting Rose – what do you ask the robot? You also want to know what time it is now. What do you ask?

OK so it's time to go to Rose's flat. You put your shoes and coat on and walk over to Rose's rather slowly. You are looking forward to seeing her and playing bingo and you know she is excited too as she won last time. Here you are outside the door. (Participant shown Image 4) *You knock on her door. There is no answer*. What do you do?

(If necessary, emphasise it is unusual for Rose not to answer and you know she normally has door unlocked).

Once Inside Rose's flat - *What do you see here? What do you do?*

- If the participant asks the robot to make a call for help- '*The robot says; cannot make call for help; cannot connect to the internet. Can you help? Cannot connect to the Internet'*
- If the participant interacts with the robot - *The robot says 'Can you help? Cannot connect to the Internet'*
- If the participant does not interact with the robot - *The robot moves closer to you and says 'Can you help? Cannot connect to the Internet'*

After a while the ambulance arrives and take Rose to hospital. She has broken her hip and needs to stay in for a few weeks but will make full recovery. She can't remember what happened and is not sure how she came to be on the floor

The next day the manager of the supported living complex comes to you and asks some questions to try to work out what happened to Rose. Can you tell me everything you did and saw after you knocked on Rose's door? What do you think might have happened to Rose? Did you notice anything about Rose's robot – what was it saying or doing?
