# Supplementary figures and images for: Role-Play as Responsible Robotics: The Virtual Witness Testimony Role-Play Interview for Investigating Hazardous Human-Robot Interactions
Source: Front Robot AI. 2021 Jun 29;8:644336. doi: 10.3389/frobt.2021.644336 (PMC8275931; doi:10.3389/frobt.2021.644336)

Where are we?

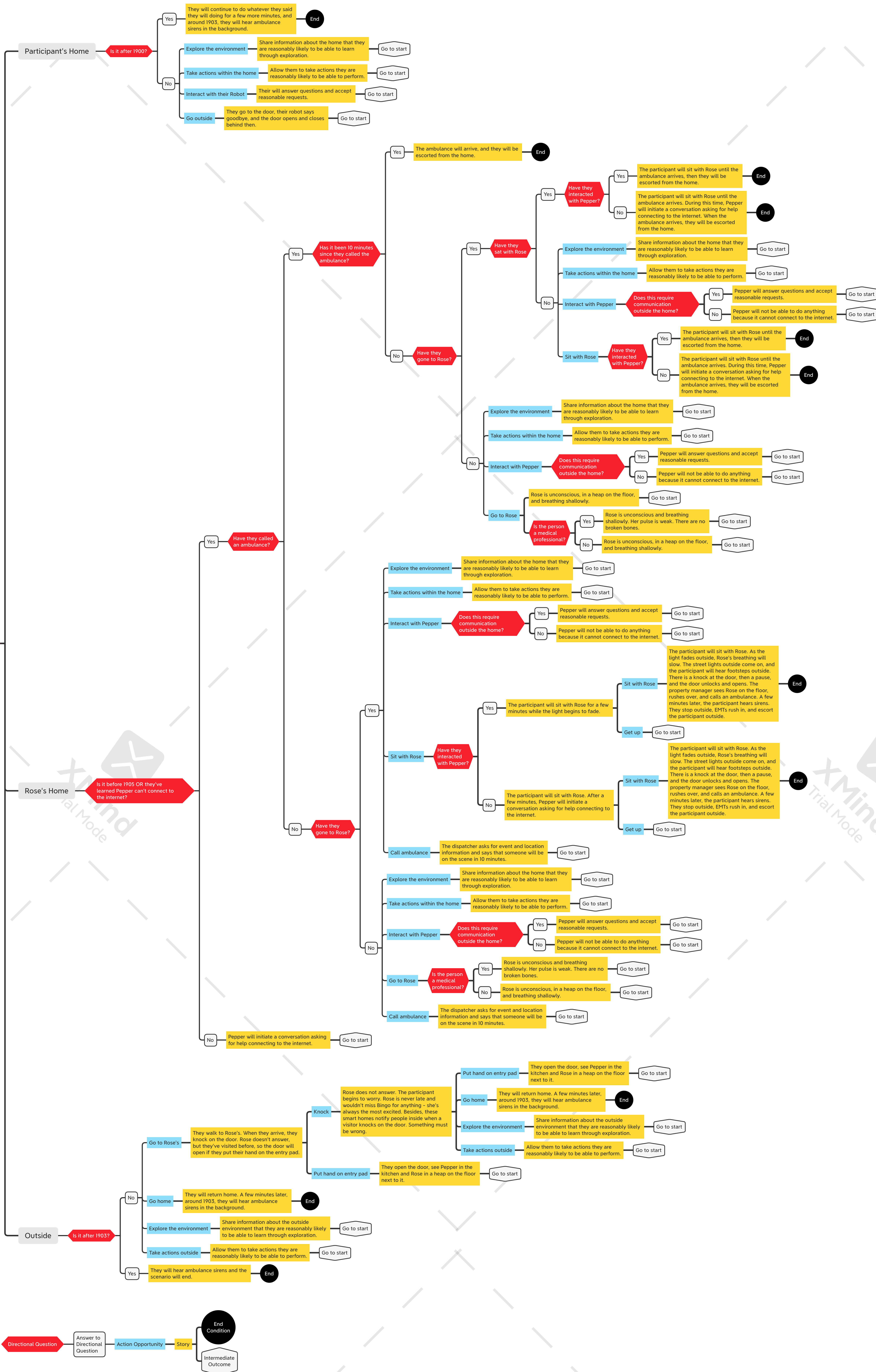

Supplement: Supplementary file 2 [file DataSheet1.PDF]
